# Design Matters? Evaluating Implant Macro‐Geometry in Immediate Implants: A Randomized Clinical Trial With Six‐Month Interim Analysis

**DOI:** 10.1002/cre2.70490

**Published:** 2026-10-25

**Authors:** Paolo Carosi, Andrea Laureti, Abdusalam Alrmali, Alessandro Pinto, Hom‐Lay Wang, Claudia Lorenzi

**Affiliations:** ^1^ CL Dentistry Segni Italy; ^2^ Department of Periodontics and Oral Medicine, School of Dentistry University of Michigan Ann Arbor Michigan USA; ^3^ Division of Fixed Prosthodontics and Biomaterials University Clinics for Dental Medicine, University of Geneva Geneva Switzerland; ^4^ Department of Oral Pathology, Oral Medicine, and Oral & Maxillofacial Surgery University of Tripoli School of Dentistry Tripoli Libya; ^5^ Private practice Salerno Italy

**Keywords:** early healing, immediate implants, implant macrodesign, implant stability quotient, osseointegration, randomized controlled trial, resonance frequency analysis

## Abstract

**Objectives:**

To compare the early stability trajectories of two tissue‐level implant macro‐designs after immediate placement, using monthly resonance frequency analysis (ISQ) over 6 months.

**Materials and Methods:**

Single‐center randomized clinical trial in adults requiring immediate implant placement in posterior sites. ISQ was recorded at implant placement and monthly for 6 months. A linear mixed‐effects model (subject‐level random intercept; fixed effects for group, visit, their interaction, and baseline ISQ) was fitted, with the group × visit interaction as the primary inferential term. The 6‐month endpoint was assessed by baseline‐adjusted ANCOVA, with equivalence tested against a ±3 ISQ margin (2 one‐sided tests).

**Results:**

Thirty implants were placed in 30 patients (aggressive self‐tapping, *n* = 15; conservative uniform‐thread, *n* = 15). ISQ increased significantly over time in both groups (*p* < 0.001). The group × visit interaction was significant (χ^2^ = 43.25, df = 5, *p* < 0.001); baseline‐adjusted differences favored the aggressive design at t1 (+3.84 ISQ, 95% CI 1.64 to 6.03; *p* = 0.004) and t2 (+3.70, 95% CI 1.51 to 5.90; *p* = 0.005), and were no longer detectable from t3. At 6 months, the baseline‐adjusted difference was −0.73 ISQ (95% CI −2.91 to 1.46; *p* = 0.500), and equivalence within ±3 ISQ was supported (TOST *p* = 0.021). All aggressive‐design implants reached ISQ ≥ 70 by 3 months versus 33% in the conservative group (*p* = 0.001).

**Conclusions:**

Within the limitations of this trial, the more aggressive macro‐design showed significantly higher stability during the first 2 months of healing, with significantly different trajectories over time, whereas at 6 months, no between‐group difference was demonstrated and equivalence within the pre‐specified ±3 ISQ margin was supported. Macro‐design therefore influenced the timing of stability acquisition rather than stability at 6 months, supporting its relevance for early loading protocols.

**Trial registration:** ClinicalTrials.gov identifier: NCT06711744; Date of registration: 21 November 2024; EC Protocol ID: 6271 (Ethics Committee Lazio 3).

## 1 Introduction

Immediate implant placement requires the rapid establishment of implant stability to support predictable healing and, where indicated, early loading. That stability derives from osseointegration, the direct functional and structural connection between newly formed bone and the surface of a load‐bearing implant, which is influenced by both implant‐related and patient‐related factors, including macro‐ and micro‐design characteristics (Albrektsson et al. 1981; Buser et al. 2012, 2017; Davies 1998; Di Gianfilippo, Chambrone et al. 2022; Pradyachaipimol et al. 2023; Schroeder et al. 1976, 1981).

Implant macrodesign (e.g., body shape, thread geometry, and collar configuration) plays a key role in mechanical retention, primary stability, and strain distribution at the bone–implant interface (Alves and Neves 2009; Carosi et al. 2023; Lang et al. 2007; Sugiura et al. 2019; Wilson Jr et al. 2013). Microdesign features, such as surface topography and chemistry, may further enhance early biological events and accelerate osseointegration (Abrahamsson et al. 2004; Buser et al. 1991, 2004; Hickok et al. 2018; Lang et al. 2011; Raghavendra et al. 2005; Schwartz and Boyan 1994). Because early micromotion may compromise osseointegration, achieving sufficient mechanical stability at placement remains critical (Berglundh et al. 2003; Raghavendra et al. 2005). Primary stability is commonly assessed using insertion torque and resonance frequency analysis (RFA), reported as implant stability quotient (ISQ) values (Meredith 1998). RFA enables non‐invasive monitoring over time, although the relationship between insertion torque, ISQ values, and healing dynamics is still debated (Lages et al. 2018; Levin 2016; Meredith 1998; Meredith et al. 1997; Rasmusson et al. 1997; Sanz‐Sánchez et al. 2015; Schliephake et al. 2006; Sennerby and Meredith 2008).

Despite the widespread clinical use of RFA, longitudinal ISQ data in immediate implant placement remain limited, particularly for newer tissue‐level macrodesigns and specific anatomical scenarios (Atieh et al. 2018; Bergamo et al. 2021; Khandelwal et al. 2013; Kim et al. 2015; Malchiodi et al. 2016; Marchio et al. 2023; McCullough and Klokkevold 2017; Simunek et al. 2012; Torroella‐Saura et al. 2015). Therefore, this randomized controlled trial was designed to characterize the early healing dynamics of implant stability rather than a medium‐term endpoint. Its primary aim was to determine whether two tissue‐level implant macro‐designs placed immediately after extraction follow different stability trajectories during the first 6 months, assessed by monthly RFA. The rationale for this focus is that macro‐geometry exerts its mechanical effect at placement and during the transition from primary to secondary stability, so any influence of design is expected to be greatest in the early healing period and to diminish as osseointegration progresses. Secondary aims were to quantify baseline‐adjusted between‐group differences at each visit, to determine when each design reached the stability level conventionally considered compatible with loading, and to explore associations between stability and implant‐ and patient‐related variables, including native bone engagement. The null hypothesis was that the two designs would show identical stability trajectories over the observation period.

## 2 Materials and Methods

### 2.1 Study Design

This single‐center randomized controlled trial with longitudinal repeated ISQ assessment was approved by the Ethics Committee Lazio 3, Rome, Italy (Protocol ID 6271) and registered at ClinicalTrials.gov (NCT06711744). It was reported in accordance with CONSORT and conducted in compliance with the Declaration of Helsinki and ICH E6(R2) Good Clinical Practice (Figure 1). All participants gave written informed consent. The registered protocol specifies ISQ monitoring through 12 months; the present report is a pre‐planned interim analysis at 6 months.

**Figure 1:**
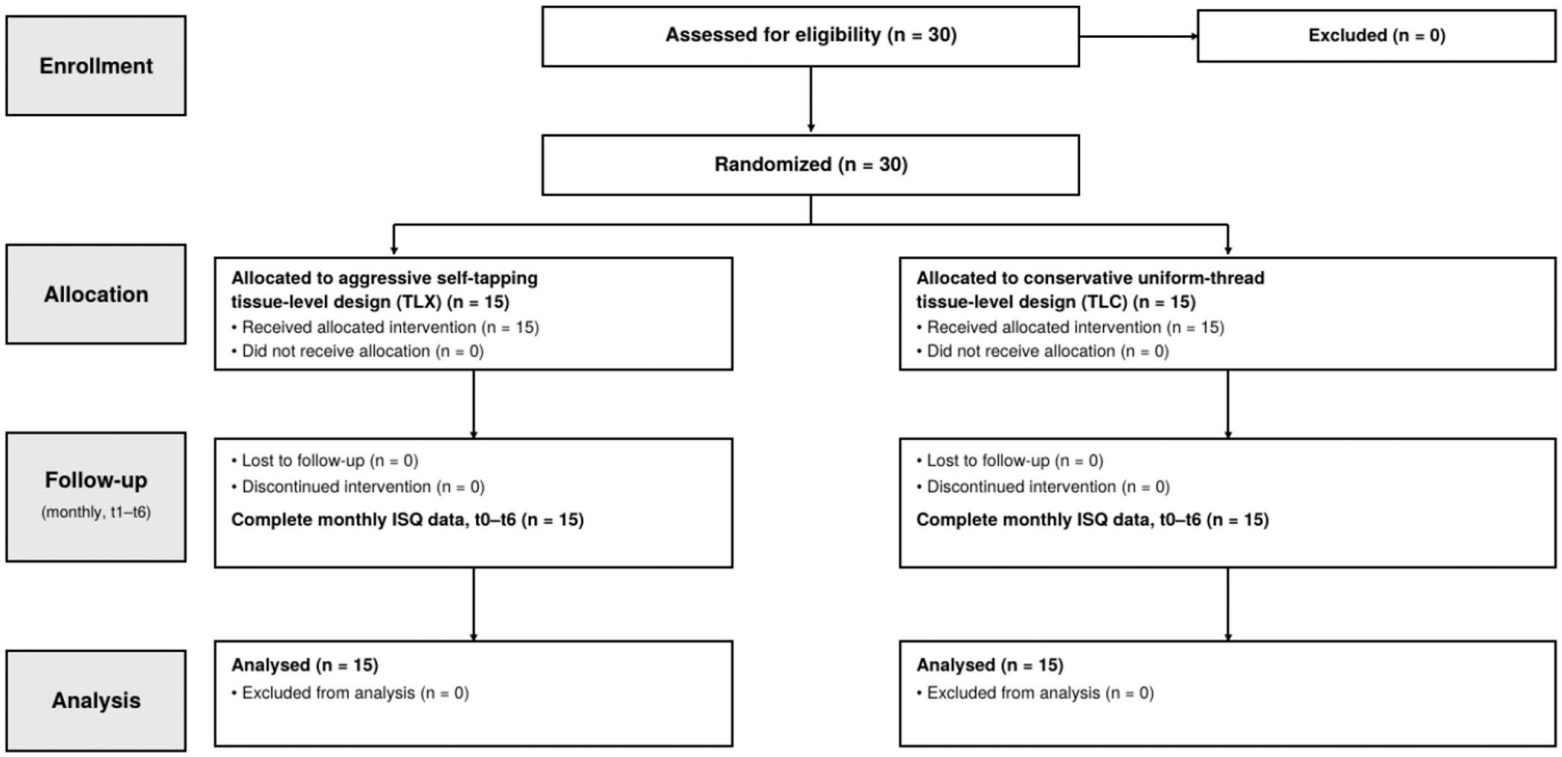
CONSORT flow diagram.

### 2.2 Study Population

Patients requiring immediate replacement of a molar or premolar were consecutively enrolled and treated at a single private clinic (CL Dentistry, Segni, Italy) between January and June 2025. Eligible patients were systemically healthy, had a clinical indication for implant placement, a fresh extraction socket with at least 4 mm of residual bone beyond the root apex, adequate bone height for an implant of at least 8 mm, and sufficient width for a 3.75 mm diameter implant, and were able to attend monthly follow‐up for 6 months. Exclusion criteria comprised ASA class III or higher, psychiatric contraindications, pregnancy or nursing, medication affecting bone remodeling, alcohol or drug abuse, smoking of more than 10 cigarettes daily, radiotherapy, untreated periodontitis, acute or chronic infection of adjacent teeth, severe skeletal discrepancy, and rotational instability at placement. The full eligibility criteria are provided in the supplementary material. All participants gave written informed consent.

### 2.3 Randomization, Allocation Concealment, and Blinding

Participants were randomly allocated 1:1 to the TLC (test) or TLX (control) implant. An independent investigator (AL), not involved in recruitment, treatment, follow‐up, or analysis, generated the sequence by computer‐generated block randomization (block size 4), with allocation concealed in sequentially numbered opaque sealed envelopes opened immediately before placement. The trial was single‐blind: the surgeon was necessarily aware of allocation, whereas the examiner performing all ISQ measurements (CL) was blinded throughout, which was feasible because both systems share the same SmartPeg, transmucosal collar, and prosthetic connection and are distinguishable only radiographically. Allocation codes were disclosed only after data entry and analysis were complete.

### 2.4 Intervention and Control

Each participant underwent cone beam computed tomography (ProXam 3DQ Pro, Kavo, Kloten, Switzerland) and an intraoral scan, or digitization of a conventional cast (D2000, 3Shape, Copenhagen, Denmark). Data sets were merged in implant planning software (coDiagnostix, Dental Wings, Montreal, Canada) for prosthetically driven planning, at which stage planned engagement with native bone was quantified as the linear apical bone contact in millimeters.

Implants were placed in an ideal three‐dimensional position by a single experienced clinician (PC) using a tooth‐ and mucosa‐supported surgical stent derived from the planning data; no intraoperative CBCT was performed. The TLX is a fully tapered tissue‐level implant with a self‐cutting, self‐tapping macro‐design and a condensing body, a titanium‐zirconium alloy core (Roxolid) and a hydrophilic sandblasted, acid‐etched surface (SLActive), with a 1.8 mm machined collar (TLX SP, Institut Straumann AG, Basel, Switzerland) (Figure 2).

**Figure 2:**
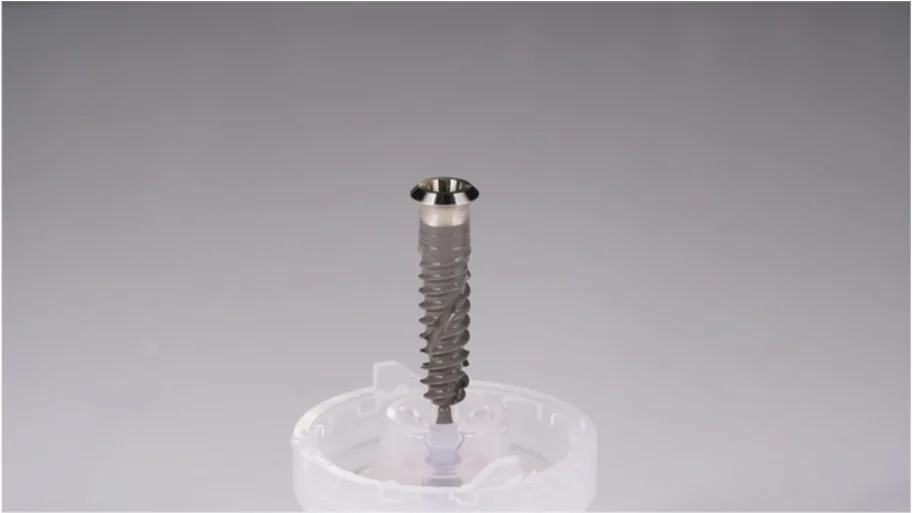
Control implant (TLX SP, Institut Straumann AG, Basel, Switzerland).

The TLC implant is a tissue‐level tapered implant characterized by a tapered apex, a uniform thread pitch with less aggressive thread geometry, and a condensing body. Like the TLX, it features a Roxolid® core and an SLActive® surface, with a smooth, machined collar measuring 1.8 mm in height (TLC SP, Institut Straumann AG, Basel, Switzerland) (Figure 3).

**Figure 3:**
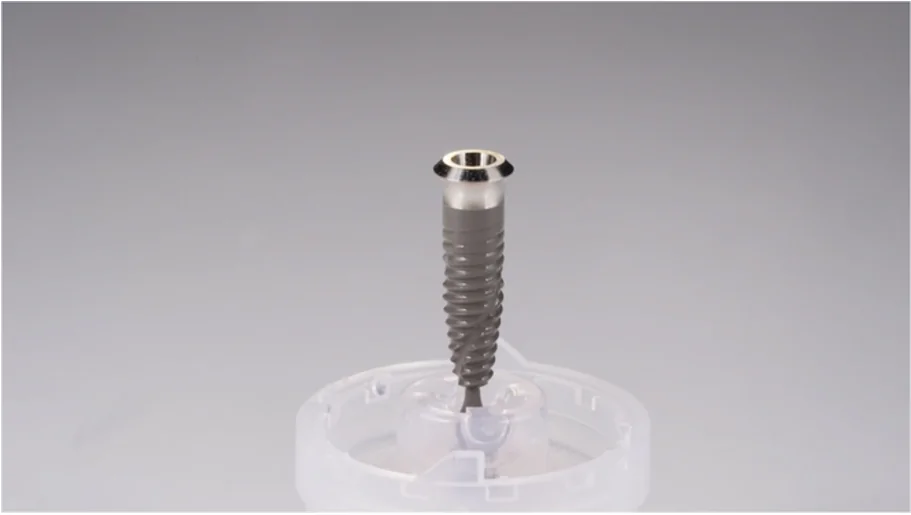
Test implant (TLC SP, Institut Straumann AG, Basel, Switzerland).

Following implant placement, insertion torque (IT) and ISQ values were recorded. All implants healed transmucosally, and healing abutments were tightened to 10 Ncm. Representative clinical cases are shown for a TLC implant immediately placed in a molar site (Figure 4) and a TLX implant immediately placed in a premolar site (Figure 5). Flap closure was achieved using single‐interrupted, internal mattress, or sling sutures (5‐0 Vicryl, Ethicon, Somerville, NJ, USA).

**Figure 4:**
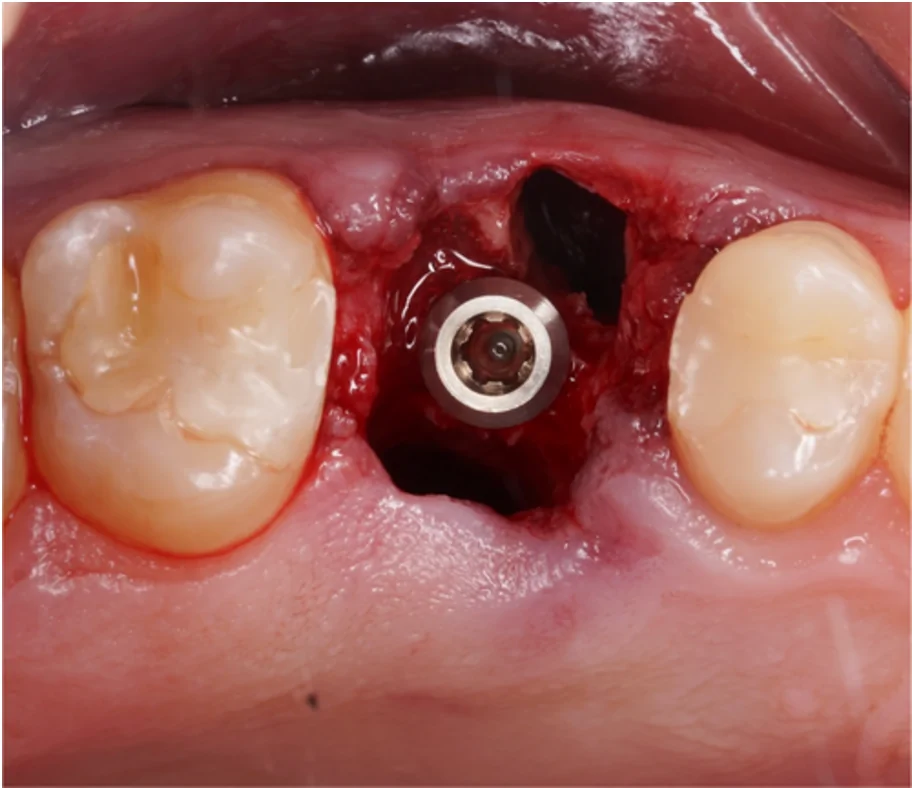
TLC implant immediately placed in molar site.

**Figure 5:**
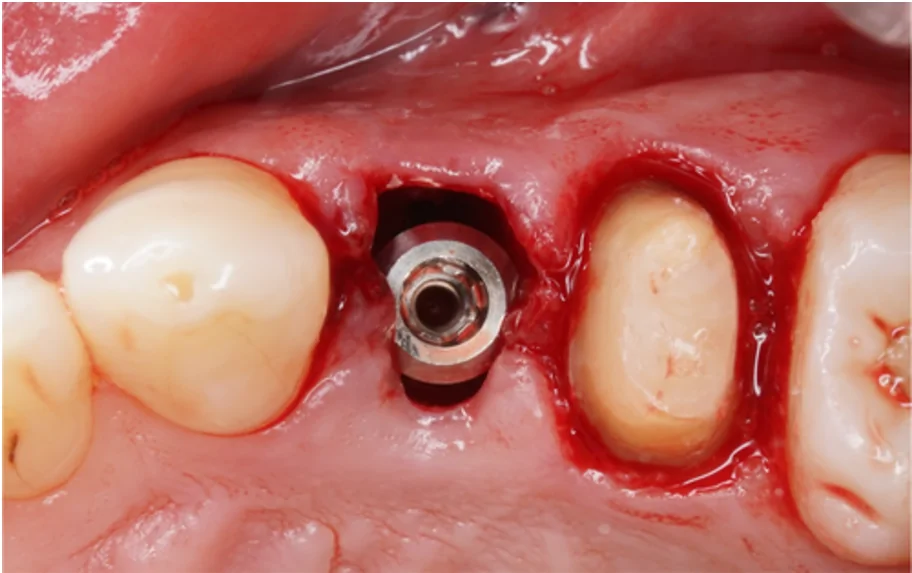
TLX implant immediately placed in premolar site.

Postoperative instructions comprised twice‐daily 0.2% chlorhexidine rinses, a soft diet, and avoidance of trauma for 1 month. An early loading protocol was applied: provisional resin crowns were delivered 2 months after placement and definitive screw‐retained metal‐ceramic restorations at 4 months. Measurements from t2 onward therefore reflect a functionally loaded environment. Loading was applied identically in both groups by protocol and was not analyzed as an explanatory variable; it is considered a potential time‐dependent factor affecting the later observation period rather than an explanation for the observed trajectories.

Adjunctive procedures were performed according to pre‐specified intraoperative criteria. Where the horizontal gap between the implant surface and the inner aspect of the buccal plate exceeded 2 mm, the gap was grafted with demineralized bovine bone material with collagen (Xenoflex, Institut Straumann AG, Basel, Switzerland). A connective tissue graft was harvested from the palate and positioned in a supraperiosteal envelope where the buccal soft‐tissue thickness was below 2 mm, in order to compensate for the expected post‐extraction contour change. No barrier membrane was used. Socket grafting combined with a connective tissue graft was performed in 22 sites, socket grafting alone in 5 and a connective tissue graft alone in 3; the distribution by group is reported in Table 1.

**Table 1:** Descriptive analysis of implant stability quotient (ISQ) values for TLX and TLC implants, measured monthly during the 6‐month follow‐up. Data are presented as minimum, maximum, mean, standard deviation, median, and 25th–75th percentiles for insertion torque and for ISQ at each visit (t0–t6).

	TLC							TLX						
	Min	Max	Mean	SD	Median	Percentile 25	Percentile 75	Min	Max	Mean	SD	Median	Percentile 25	Percentile 75
INS TORQ	15.0	50.0	28.7	10.4	30.0	22.5	35.0	15.0	60.0	37.0	14.2	40.0	25.0	50.0
ISQ t0	48.0	65.0	54.7	4.7	55.0	50.0	58.0	42.0	73.0	63.7	7.9	65.0	60.5	68.5
ISQ t1	50.0	66.0	59.5	4.2	60.0	57.0	62.0	54.0	72.0	65.7	5.2	66.0	61.5	70.0
ISQ t2	59.0	70.0	63.9	3.8	63.0	61.0	67.0	60.0	74.0	70.0	3.5	70.0	68.5	73.0
ISQ t3	64.0	75.0	68.6	2.9	69.0	67.0	70.0	70.0	75.0	72.0	2.2	72.0	70.0	74.0
ISQ t4	67.0	77.0	71.7	3.1	72.0	70.0	74.0	70.0	77.0	73.5	2.5	73.0	72.0	75.0
ISQ t5	69.0	77.0	73.5	2.7	75.0	71.0	75.0	69.0	80.0	74.4	2.8	75.0	72.5	76.0
ISQ t6	71.0	79.0	74.9	2.4	75.0	73.5	76.5	70.0	78.0	74.9	2.3	75.0	74.0	76.5

#### 2.4.1 ISQ Monthly Assessment

Healing abutments were removed monthly to attach high‐precision metal SmartPegs (Type 47, Osstell, Gothenburg, Sweden) to the implants. Implant stability was then evaluated by a single experienced examiner (CL) using an RFA device (Osstell IDx, Osstell, Gothenburg, Sweden).

ISQ measurements were recorded in two directions (bucco‐lingual and mesio‐distal) at each visit; the mean of the two readings was used for analysis. If the two directional readings differed by more than two ISQ units, a third measurement was performed and the mean of all readings was recorded. Assessments were carried out at implant placement (t0) and monthly for 6 months (t1–t6).

The examiner had prior experience with RFA and followed a standardized measurement protocol throughout, with the SmartPeg hand‐tightened to the manufacturer's recommended torque and the probe held perpendicular to the SmartPeg at a constant distance. No formal intra‐examiner reproducibility study was undertaken, and this is acknowledged as a limitation.

Peri‐implant diagnostic evaluations were performed every 3 months following Di Gianfilippo, Askar, et al. (2022) with supportive periodontal therapy. Restorative screws were replaced at the last study visit (t6).

### 2.5 Outcomes

The primary outcome was the ISQ, recorded at implant placement (t0) and at each monthly visit thereafter to 6 months (t1–t6). The primary estimand was the difference between groups in the ISQ trajectory over time, operationalized as the group × visit interaction in a baseline‐adjusted linear mixed‐effects model.

Secondary outcomes were the baseline‐adjusted between‐group difference in ISQ at each visit; the difference at the 6‐month endpoint; and the proportion of implants at or above ISQ 70, the value conventionally regarded as compatible with early loading. Exploratory outcomes were the association between engagement with native bone and ISQ, and the influence of implant length, site, and sex on the trajectory. The change score (ΔISQ = ISQ t6 − ISQ t0) is reported descriptively only.

### 2.6 Statistical Analysis

Sample size was calculated using G*Power software (Heinrich Heine University, Düsseldorf, Germany) for a repeated‐measures ANOVA design with one between‐subject factor (implant group: TLX vs. TLC). The calculation was based on longitudinal ISQ data from a previously published 1‐year prospective study on tissue‐level implants of the same system, which reported a partial η^2^ of 0.611 for the time effect, corresponding to a Cohen's *f* of approximately 1.25, and a mean correlation between repeated measures of 0.75 (Carosi et al. 2025). Setting α = 0.05, power = 0.80, seven repeated measurements (t0–t6), and a Greenhouse–Geisser epsilon of 0.20 to account for sphericity violation, a minimum of 13 implants per group was estimated. Fifteen implants per group were enrolled to allow for potential dropouts. Because the study protocol and sample size calculation predated the current statistical framework, the original repeated‐measures ANOVA assumptions were retained for the a priori sample size estimation; the adequacy of the sample for the secondary between‐group analyses is addressed in the limitations.

All analyses were performed with IBM SPSS Statistics (version 30). Continuous variables are presented as mean ± standard deviation and categorical variables as counts (%). Normality was assessed using the Shapiro–Wilk test. Statistical significance was set at *p* < 0.05 and all tests were two‐sided.

Longitudinal ISQ data were analyzed using a baseline‐adjusted linear mixed‐effects model fitted to the six post‐baseline visits, with fixed effects for group, visit (categorical), the group × visit interaction, and baseline ISQ, and a subject‐level random intercept. The group × visit interaction was tested by likelihood‐ratio test comparing nested models fitted by maximum likelihood. The final model was then refitted by restricted maximum likelihood for estimation of visit‐specific contrasts and confidence intervals. The group × visit interaction was the primary inferential term, tested by likelihood‐ratio test. Given the sample size, the fixed‐effects structure was deliberately parsimonious; sex, site, and implant length were each examined singly in pre‐specified sensitivity models (supplementary material).

Visit‐specific between‐group differences were estimated as model‐based contrasts with 95% confidence intervals and adjusted across the six post‐baseline visits using the Holm–Bonferroni procedure; unadjusted comparisons with Cohen's *d* are also reported. The 6‐month endpoint was analyzed by analysis of covariance (ANCOVA) with baseline ISQ as covariate; this endpoint ANCOVA is reported separately from the visit‐specific mixed‐model contrast at t6, because the two analyses estimate the between‐group difference under different model assumptions and are not interchangeable. Equivalence at the endpoint was assessed by two one‐sided tests (TOST) against a pre‐specified ±3 ISQ margin, with the corresponding 90% confidence interval reported; equivalence was considered demonstrated only if that interval lay entirely within the margin. The proportion of implants reaching ISQ ≥ 70 was compared at each visit by Fisher's exact test with Holm adjustment. The change score (ΔISQ = ISQ t6 − ISQ t0) is reported descriptively, because change scores are mathematically coupled to their own baseline; no inference is drawn from the unadjusted ΔISQ. The change score (ΔISQ = ISQ t6 − ISQ t0) was reported descriptively because it is mathematically coupled to baseline ISQ. Inferential conclusions regarding between‐group differences were based on the baseline‐adjusted longitudinal model and the prespecified 6‐month endpoint ANCOVA. The single baseline‐adjusted estimate reported for the 6‐month endpoint therefore applies to both parameterizations, and all inference is based on baseline‐adjusted models. Pearson correlations were used for the exploratory analyses of native bone engagement.

## 3 Results

Thirty participants (18 women, 12 men; mean age 57.1 ± 11.6 years) received 30 implants of 3.75 mm diameter, 15 per group, and all completed 6‐month follow‐up. Nine implants were placed in molar and 21 in premolar sites, and 19 in the maxilla and 11 in the mandible. Socket grafting with a connective tissue graft was performed in 22 sites, socket grafting alone in 5, and a connective tissue graft alone in 3. Groups were comparable for sex, implant site and arch (TLX 3 molar/12 premolar, 10 maxillary/5 mandibular; TLC 6 molar/9 premolar, 9 maxillary/6 mandibular) (all *p* > 0.05), but differed in implant length (11.5 ± 1.2 mm vs. 10.1 ± 1.8 mm; *p* = 0.022) and in baseline ISQ (63.7 ± 7.9 vs 54.7 ± 4.7; *p* < 0.001); both imbalances were treated as chance findings and handled by covariate adjustment. Insertion torque was numerically higher for TLX implants but did not differ significantly (*p* = 0.078). Baseline characteristics are reported in Supporting Information S1: Table S1.

### 3.1 Primary Outcome: Longitudinal ISQ Evolution

ISQ increased significantly throughout follow‐up in both groups (*p* < 0.001), reaching 74.9 in both groups at 6 months (Table 1). Within‐group Holm‐adjusted comparisons showed significant increases at every interval in the TLC group, whereas in the TLX group, increases were confined to the t1–t2 and t3–t4 intervals, indicating earlier stabilization.

Longitudinal ISQ trajectories are shown in Figure 6. Both groups showed progressive stabilization, with the TLX group higher during early healing and the two groups converging thereafter. In the linear mixed‐effects model, the group × visit interaction was highly significant (likelihood‐ratio χ^2^ = 43.25, df = 5, *p* < 0.001), indicating that the designs followed different stability trajectories and that the between‐group difference was not constant over time.

**Figure 6:**
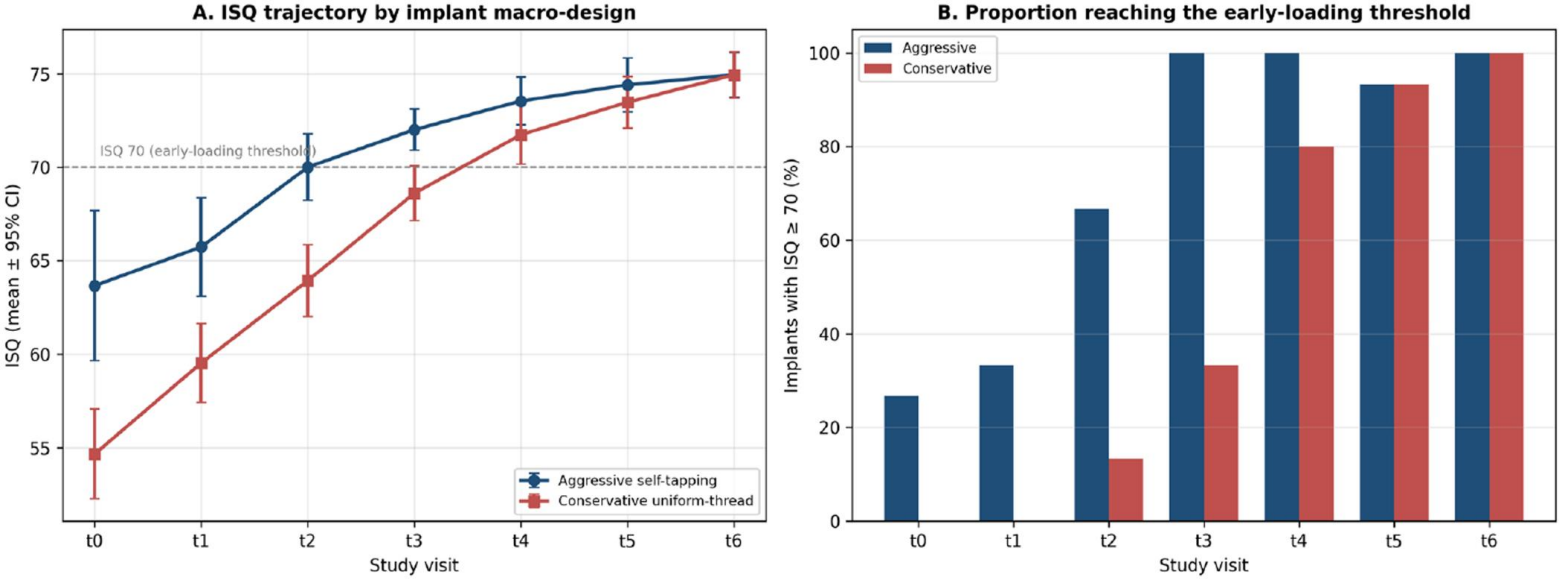
(A) Longitudinal changes in mean ISQ (±95% CI) over 6 months for the TLX and TLC groups; asterisks mark visits at which the baseline‐adjusted between‐group difference remained significant after Holm correction. TLX values were higher during early healing and the two groups converged from t4 onward, with comparable means at t6. (B) Proportion of implants reaching ISQ ≥ 70, the value conventionally regarded as compatible with early loading, at each visit.

Baseline ISQ predicted subsequent values (*B* = 0.26, 95% CI 0.16 to 0.36; *p* < 0.001) and the subject‐level random intercept accounted for 29% of residual variance. Diagnostics were satisfactory and a sensitivity analysis excluding one low baseline observation did not alter any conclusion. Subgroup analyses showed comparable ISQ trajectories throughout the follow‐up period between male and female patients (*p* = 0.118) and between maxillary and mandibular sites (*p* = 0.243) (Supporting Information S1: Figures S1 and S2). In pre‐specified single‐covariate sensitivity models, adding implant length, arch or tooth type to the baseline‐adjusted mixed model did not materially alter the between‐group effect (+0.50 to +0.74 ISQ units, vs. +0.70 in the primary model; all *p* > 0.05). These analyses are exploratory and do not replace the primary analysis.

The overall change from baseline to 6 months was greater in the TLC group (Table 1). This descriptive difference is not interpretable as a design effect: baseline ISQ and subsequent change correlated at −0.955 (*p* < 0.001). After adjustment for baseline, implant design had no significant effect at the 6‐month endpoint (adjusted difference −0.73 ISQ units, 95% CI −2.91 to 1.46; *p* = 0.500); because ANCOVA on ΔISQ and on t6 share the same group coefficient, this single estimate describes both parameterizations. The larger unadjusted ΔISQ in the conservative group therefore reflects its lower starting value and does not indicate greater absolute stability, since absolute ISQ at 6 months was the same in both groups. No inference is drawn from this quantity.

### 3.2 Secondary Outcome: Timepoint‐Specific ISQ Differences and Implant Engagement With Native Bone

Baseline‐adjusted contrasts showed significantly higher ISQ for TLX implants at t1 (+3.84, 95% CI 1.64 to 6.03; Holm‐adjusted *p* = 0.004) and t2 (+3.70, 95% CI 1.51 to 5.90; *p* = 0.005), with no significant difference from t3 onward. In unadjusted comparisons, differences were significant at t1, t2, and t3 after Holm correction, with large effect sizes (Cohen's *d* 1.32 to 1.65), and were not significant at t4 or t5. At 6 months, the unadjusted mean ISQ was 74.9 in both groups (*d* = 0.00). In the pre‐specified endpoint ANCOVA, the baseline‐adjusted between‐group difference (TLX − TLC) was −0.73 ISQ units (95% CI −2.91 to 1.46; *p* = 0.500), with a baseline covariate coefficient of 0.08 (95% CI −0.06 to 0.22; *p* = 0.250). Equivalence within the pre‐specified ±3 ISQ margin was supported: both one‐sided tests were significant (*p* = 0.021 and *p* < 0.001; TOST *p* = 0.021) and the 90% confidence interval for the adjusted difference (−2.54 to 1.09) lay entirely within the margin. Visit‐specific estimates are given in Table 2 and Figure 6.

**Table 2:** Baseline‐adjusted between‐group differences in ISQ at each post‐baseline visit, estimated as model‐based contrasts from the linear mixed‐effects model. Estimates are model‐based contrasts from the linear mixed‐effects model, not independent‐samples *t*‐tests; positive values indicate higher ISQ in the TLX (aggressive self‐tapping) group relative to TLC (conservative uniform‐thread), which is the reference group. The t6 contrast shown here is not the pre‐specified endpoint analysis: the endpoint estimate is derived from the ANCOVA reported in Section Results, 3, and the two differ for the reasons given there. *p* values were adjusted for multiplicity across the six post‐baseline visits using the Holm–Bonferroni procedure.

Visit	Adjusted difference, ISQ units (95% CI)	*p* (nominal)	*p* (Holm‐adjusted)	Cohen's *d* a
t1	+3.84 (1.64 to 6.03)	< 0.001	0.004*	1.32
t2	+3.70 (1.51 to 5.90)	0.001	0.005*	1.65
t3	+1.04 (−1.16 to 3.23)	0.355	0.711	1.33
t4	−0.56 (−2.76 to 1.63)	0.614	0.711	0.63
t5	−1.43 (−3.63 to 0.76)	0.201	0.604	0.34
t6	−2.36 (−4.56 to −0.17)	0.035	0.139	0.00

^a^ Cohen's *d* from unadjusted between‐group comparisons, reported descriptively.

* Statistically significant differences after adjustment.

The visit‐specific mixed‐model contrast at t6 (−2.36 ISQ units, 95% CI −4.56 to −0.17; nominal *p* = 0.035; Holm‐adjusted *p* = 0.139; Table 2) differs from the endpoint ANCOVA estimate (−0.73 ISQ units) because the two models estimate the between‐group difference under different model assumptions. The endpoint ANCOVA was the pre‐specified analysis for the 6‐month comparison and was therefore used for the endpoint equivalence assessment.

Exploratory analyses showed no significant association between planned engagement with native bone and either baseline ISQ or the overall change in ISQ, in the TLX group (*r* = 0.118, *p* = 0.676 and *r* = −0.204, *p* = 0.466) or the TLC group (*r* = 0.346, *p* = 0.175 and *r* = 0.320, *p* = 0.245).

### 3.3 Time to Reach ISQ ≥ 70

The proportion of implants reaching ISQ ≥ 70, a commonly used descriptive reference value, differed markedly during early healing: 10/15 (67%) versus 2/15 (13%) at t2 (Holm‐adjusted *p* = 0.047) and 15/15 (100%) versus 5/15 (33%) at t3 (*p* = 0.001) for TLX and TLC, respectively. By t4 the proportions were 15/15 and 12/15, and no statistically significant between‐group difference was detected at t5 or t6, with all implants at or above the threshold at t6. Every TLX implant had therefore reached ISQ ≥ 70 by 3 months, whereas in the TLC group this proportion was reached between 5 and 6 months. ISQ ≥ 70 is a commonly used reference value rather than a validated threshold for loading; it was not pre‐specified as a clinical decision rule in this trial, and this comparison is descriptive. Reaching this value does not by itself establish readiness for loading (Figure 6B).

### 3.4 Implant Survival and Adverse Events

All 30 implants were available for evaluation at every scheduled visit; no participant was lost to follow‐up and no implant was excluded from analysis. Implant survival, defined as the presence of the implant in situ and functionally loaded at the final study visit, was 100% in both groups. No biological or mechanical complications and no serious adverse events attributable to the study procedures were recorded in either group during the observation period. Formal implant success criteria were not pre‐specified and were not assessed; peri‐implant clinical parameters will be reported with the 12‐month data set.

### 3.5 Analysis Population and Protocol Deviations

All analyses were performed on an intention‐to‐treat basis, including all 30 randomized participants according to their allocated group. No protocol deviations were recorded, no participant crossed over between groups, and no imputation was required, since the data set was complete at every visit.

## 4 Discussion

This randomized controlled trial compared the early stability trajectories of two tissue‐level implant macro‐designs placed immediately after extraction, with monthly RFA over 6 months.

The principal finding is that the two macro‐designs differed substantially in stability during the first 2 to 3 months of healing and did not differ at the 6‐month endpoint. Both elements are findings, and the second is not a failure of the first. The formal group × visit interaction confirmed that the trajectories genuinely diverged (*p* < 0.001). The baseline‐adjusted comparison at 6 months did not demonstrate a statistically significant between‐group difference, and the pre‐specified equivalence test supported equivalence within a ±3 ISQ margin. These findings indicate that the level of stability attained by 6 months did not differ detectably between the two macro‐designs within that margin; they do not establish that stability is independent of macro‐geometry in a more general sense. Macro‐design therefore influenced the timing of stability acquisition rather than the stability ultimately achieved, and the question it informs is when an implant may be loaded rather than how stable it will ultimately become.

The higher ISQ values recorded for the TLX design during early healing are plausibly explained by its tapered, self‐cutting macro‐geometry, which promotes greater mechanical interlocking at placement. Both designs nevertheless converged by 6 months, indicating that implants with lower initial mechanical retention achieve comparable secondary stability within the observation period. After baseline adjustment and correction for multiplicity, differences were confined to the first 2 months, consistent with macro‐design influencing the mechanically driven phase of stabilization while the biological phase proceeds comparably in both designs.

Because change scores are intrinsically influenced by baseline values, ΔISQ was considered descriptive only. In the baseline‐adjusted endpoint analysis—which, as noted in the Methods, yields the same group coefficient whether fitted to t6 or to ΔISQ—implant design had no significant effect (−0.73 ISQ units, 95% CI −2.91 to 1.46; *p* = 0.500). A greater subsequent gain in implants with lower initial stability has previously been reported as a function of the baseline value itself (Simunek et al. 2010). ΔISQ is accordingly reported as a descriptive change measure and is not interpreted as evidence of greater absolute stability.

The clinical interpretation of between‐group differences in ISQ requires equal caution, since no minimal clinically important difference has been established for this measure and differences of a few units may fall within the resolution of the method. A more meaningful framing concerns absolute values: ISQ at or above approximately 70 is commonly used as a reference value when early loading is considered, although no validated threshold has been established. On this criterion, the difference between designs may be clinically relevant during the early phase, as every TLX implant had reached ISQ ≥ 70 by 3 months, whereas only one third of TLC implants had done so at that time, suggesting earlier attainment of this commonly used stability reference value. The TLX design may therefore be advantageous when early loading is considered, although reaching ISQ ≥ 70 alone does not establish loading readiness. Two methodological considerations qualify these findings. First, a ceiling effect may contribute to the later convergence, although both groups converged at approximately 75 ISQ units, well below the values previously reported for this system at 12 months, so it is unlikely to account for the convergence in full. Second, RFA quantifies the stiffness of the implant–bone–tissue complex and is a biomechanical surrogate rather than a direct measure of bone formation, so the mechanistic interpretations offered here are consistent with, rather than demonstrated by, our observations. Relatedly, although the mean ISQ did not fall between placement and the first post‐operative visit, this cannot be taken as evidence that no transient reduction occurred, since the reported nadir falls around the third to fourth week (Todorovic et al. 2017) and therefore coincides with our first measurement. Notably, five of fifteen TLX implants did show a decrease at t1, whereas none in the TLC group did, consistent with excessive bone compression transiently impairing the reparative response where initial stability is high.

This study has several limitations. The sample is small, and the a priori calculation was based on the longitudinal time effect rather than on the between‐group comparison; the secondary analyses should therefore be interpreted with caution, because the trial was not specifically powered for between‐group comparisons, and non‐significant findings from t3 onward should be regarded as inconclusive rather than as proof of equivalence: absence of a statistically significant difference does not establish equivalence. Formal equivalence was assessed only at the 6‐month endpoint, where it was supported within a ±3 ISQ margin; the confidence interval nonetheless spans a range of roughly three ISQ units either side of zero, and the corresponding mixed‐model contrast at t6 extends beyond the lower margin, so the residual uncertainty at this sample size is not negligible. No equivalence claim is made at any other visit. Insertion torque and adjunctive procedures were examined descriptively and no formal mediation analysis was performed, so their contribution to the baseline difference cannot be quantified. Intra‐examiner reproducibility of the ISQ measurements was not formally quantified, so the extent to which small between‐group differences exceed the measurement error of the method cannot be established. Bone quality was not recorded by any classification or quantitative measure, which is a further substantive omission in a trial concerned with primary stability. Two baseline imbalances were observed despite computer‐generated block randomization with allocation concealment—baseline ISQ and implant length—both handled by covariate adjustment; implant length neither predicted baseline ISQ nor materially altered the group effect, but residual confounding cannot be excluded at this sample size. Adjunctive regenerative and soft‐tissue procedures were unevenly distributed, and prosthetic loading commenced at 2 months, so measurements from t2 onward reflect a functionally loaded rather than an undisturbed healing environment. The monthly schedule cannot resolve events within the first four post‐operative weeks. Finally, this is a pre‐planned interim analysis at 6 months of a trial registered for 12‐month follow‐up; no stopping rule was applied, enrolment was complete before the interim analysis, and all *p* values are nominal with respect to the 12‐month primary endpoint, which will be reported separately together with peri‐implant clinical parameters.

## 5 Conclusions

Within the limitations of this trial, implants with a more aggressive macro‐design achieved significantly higher stability during the first months of healing, whereas no difference was observed at the 6‐month endpoint. Implant macro‐design influenced the timing of stability acquisition rather than the stability ultimately achieved, which supports its relevance when early loading protocols are considered.

## Author Contributions

**Paolo Carosi:** conceptualization, methodology, investigation, data curation, writing – original draft. **Andrea Laureti:** methodology, randomization and allocation, writing – review and editing. **Abdusalam Alrmali:** formal analysis, data curation, writing – review and editing. **Alessandro Pinto:** investigation, data curation, writing – review and editing. **Hom‐Lay Wang:** conceptualization, supervision, writing – review and editing. **Claudia Lorenzi:** investigation, project administration, writing – review and editing.

## Ethics Statement

The trial protocol (ClinicalTrials.gov NCT06711744) was approved by the Ethics Committee Lazio 3, Rome, Italy (Protocol ID: 6271). This study was based on the Consolidated Standards of Reporting Trials (CONSORT). It was conducted in compliance with the Declaration of Helsinki for biomedical research involving human subjects as amended in 2008 and revised in 2013 (Fortaleza, Brazil), and in accordance with industry regulations (the International Council for Harmonization Guideline for Good Clinical Practice and ISO14155).

## Conflicts of Interest

The authors declare no conflicts of interest.

## Supporting information


Supporting File

## Data Availability

The de‐identified data set supporting the findings of this study, comprising all individual ISQ measurements at every visit, insertion torque values, and baseline characteristics, will be deposited in a publicly accessible repository upon publication and is available from the corresponding author on reasonable request in the interim.
