# Supplementary material for: Design Matters? Evaluating Implant Macro‐Geometry in Immediate Implants: A Randomized Clinical Trial With Six‐Month Interim Analysis
Source: Clin Exp Dent Res. 2026 Oct 25;12(5):e70490. doi: 10.1002/cre2.70490 (PMC13696025; doi:10.1002/cre2.70490)
Supplement: Supplementary file 1 [file CRE2-12-e70490-s001.docx]

|  | | Minimum | Maximum | Mean | Standard Deviation | Median | Percentile 25 | Percentile 75 | Count | Column Valid N % |
| --- | --- | --- | --- | --- | --- | --- | --- | --- | --- | --- |
| **AGE** | | 37,0 | 76,0 | 57,1 | 11,6 | 57,0 | 48,0 | 67,0 |  |  |
| **GENDER** | F |  |  |  |  |  |  |  | 18 | 60% |
|  | M |  |  |  |  |  |  |  | 12 | 40% |
| **LENGTH** | | 8,0 | 12,0 | 10,8 | 1,6 | 12,0 | 10,0 | 12,0 |  |  |
| **SITE** | MANDIBLE |  |  |  |  |  |  |  | 11 | 36,7% |
|  | MAXILLA |  |  |  |  |  |  |  | 19 | 63,3% |

**Supplementary Table 1**. Demographic and clinical characteristics of the whole study population at baseline. Continuous variables are presented as minimum, maximum, mean, standard deviation, median and 25th–75th percentiles; categorical variables as counts (percentage). Values refer to the whole sample and are not broken down by allocated group; group-specific baseline and procedural characteristics are reported in Table 1 of the main text.

**Supplementary Figure 1**. Temporal evolution of ISQ (±95% CI) by gender. Both male and female patients exhibited similar stability trends (p = 0.118).

**Supplementary Figure 2.** Temporal evolution of ISQ (±95% CI) by implant site. Maxillary and mandibular sites demonstrated comparable progressive stability increases (p = 0.243).
